# Environmental Stress and Feed Intake in Chickens: Neuroendocrine Mechanisms and Mitigation Strategies

**DOI:** 10.3390/biology15201894

**Published:** 2026-10-20

**Authors:** Saber Y. Adam, Hosameldeen Mohamed Husien, Madumetja Cyril Mathapo, Abdelkareem A. Ahmed, Mohamed Osman Abdalrahem Essa, Mudathir Y. Abdulrahman, Thobela Louis Tyasi, Demin Cai

**Affiliations:** 1 College of Animal Science and Technology, Yangzhou University, Yangzhou 225009, China; saber@duc.edu.sd (S.Y.A.); mudathir@duc.edu.sd (M.Y.A.); 2 Biomedical Research Institute, Darfur University College, Nyala 63313, Sudan; kareemo151@gmail.com; 3 College of Veterinary Medicine, University of Albutana, Rufaa 22217, Sudan; hosamhusien@albutana.edu.sd (H.M.H.); dh23054@stu.yzu.edu.cn (M.O.A.E.); 4 Department of Agricultural Economics and Animal Production, University of Limpopo, Private Bag X1106, Sovenga, Limpopo 0727, South Africa; madumetja.mathapo@ul.ac.za; 5 Department of Veterinary Sciences, Faculty of Animal and Veterinary Sciences, Botswana University of Agriculture and Natural Resources, Gaborone P.O. Box 100, Botswana

**Keywords:** endocrinology, epigenetics, poultry welfare, climate change, corticosterone, microbiome–gut–brain axis

## Abstract

Environmental stresses negatively affect feed intake in chickens, leading to reduced growth rates and significant economic losses. This review examined the molecular mechanisms of stress-induced loss of appetite, focusing on the hypothalamic regulation of appetite-associated neuropeptides, the role of the HPA axis, elevated CORT levels, and stress-induced inflammatory cytokines that inhibit appetite-stimulating signals. Current mitigation strategies, such as cooling systems, nutritional supplements, early-life programs and genetic selection using QTL mapping, have been shown to provide varying levels of effectiveness. Limited understanding of how multiple stressors act in concert at the cellular level in natural environments is a major limitation. Furthermore, the majority of studies focus on acute stress, while low-grade chronic stress models are underrepresented despite their commercial importance. A significant shortcoming is the absence of validated non-invasive biomarkers to predict stress-induced feed reductions before production losses begin. This review examines the molecular neuroendocrine mechanisms of stress-induced anorexia and the available mitigation strategies, highlighting the disconnect between mechanistic understanding and current interventions, and emphasizing the need for mechanism-based proactive measures to sustainably improve feed intake and productivity in stressed chickens. Therefore, future research should focus on integrated multi-stress frameworks and the evaluation of the lasting epigenetic effects of early-life interventions in chickens.

## Simple Summary

Chickens raised for meat and eggs frequently consume less when exposed to excessive heat, extreme cold, or confined in overcrowded, inadequately ventilated, or excessively illuminated environments. Due to the high expense of feed and the financial losses associated with delayed growth, farmers require methods to identify these issues promptly. When a chicken experiences stress, its brain and hormones transmit signals that diminish its appetite and impede digestion, although the precise mechanism remains inadequately understood. The review examines the impact of stress on the brain region responsible for appetite regulation, as well as the role of the body’s stress hormone system. Contemporary remedies like feed supplements, improved housing, and selective breeding for resilient hens primarily address stress post-damage. Comprehending these alterations in the brain and hormones may assist researchers in creating early detection mechanisms and investigating the impact of stress on chickens throughout their lifespan. Highlighting such gaps is crucial for guiding future research toward sustainable chicken production in the context of climate change.

## 1. Introduction

Chickens, an essential economic animal, supply a high-quality protein source for human nutrition. In the last ten years, the chicken industry has experienced significant advancements in breeding, precision feeding, and welfare farming [1]. Feed expenses are the principal expenditure in animal agriculture, but rising feed prices and rivalry for food and feed between humans and chickens remain a significant issue [2]. Simultaneously, the expansion of the chicken industry and population development have jointly intensified this issue. Thus, improving chicken productivity is essential for reducing the strain on feed resources amid current challenges [1]. Modern chicken breeds have achieved significant advancements in feed efficiency and production performance. The advent of gene editing technology has offered a potent method to improve the disease resistance capabilities of chickens [3]. Precision feeding has diminished feed expenses and resource wastage by employing modern technological equipment and formulating suitable feed according to the growth requirements and production objectives of chickens at various stages [4]. The advancement of intelligent technology has significantly enhanced the living conditions and raised environments for chickens, leading to healthier and more sustainable chicken products. However, the poultry industry continues to face a variety of environmental stressors even as such technologies advance.

Chicken production is significantly affected by numerous environmental stressors, resulting in substantial economic losses worldwide. Stressors involve several factors that affect the birds’ health, well-being and productivity [5]. Negative effects include suppressed appetite, decreased growth rates, decreased feed utilization efficiency and increased susceptibility to diseases, making birds more vulnerable to infections [6,7]. Heat stress (HS) is a major environmental stressor, and its severity is being exacerbated by global warming and climate change [8,9,10]. Chickens are more sensitive to the environmental stresses associated with changes in temperature because they have a high metabolic rate, generating high levels of metabolic heat. HS can occur in different levels (mild, moderate, severe) and is affected by radiation, humidity, temperature control abilities of the bird, metabolic rate, age, intensity and duration [11]. HS has serious and far-reaching consequences, affecting numerous physiological activities. Housing density is one of the most significant environmental stressors that directly impacts chickens’ health and production [12]. High stocking density may lead to increased stress, behavioral changes, and physiological alterations. Research suggests that reducing stocking density can improve wellness indicators, including reduced foot moisture and improved footpad dermatitis [12]. High stocking density is associated with increased physiological stress responses and may affect physiological, biochemical, and enzymatic parameters in the blood [13]. Although studies have indicated that young, non-cage-bound chickens may be only slightly affected by housing density stress [14], high densities generally lead to a decline in chickens’ health and performance. Cold stress is similar to heat stress, and both negatively impact chickens by affecting their stress response, such as the ratio of heterophil cells to lymphocytes, and may make them more susceptible to diseases such as necrotizing enteritis [15]. Air pollution, inadequate lighting, limited feed, water scarcity, noise pollution, and exposure to pathogens are among the most significant stressors affecting chickens’ physiology and productivity. Effective management measures are essential to mitigate the negative impacts of these factors.

Understanding the molecular interactions of chickens with environmental stressors is crucial, as these stressors directly impact feed intake, a key factor in growth, health, and overall productivity in the chicken sector. This stems from the significant economic losses and welfare problems associated with reduced feed intake during stressful conditions, particularly HS, which poses a major challenge to chicken production globally [5]. The studies show that changes in ambient temperature can influence gene expression concerning the absorption and metabolic utilization of nutrients [16]. Brain and gut neuropeptides are regulators of appetite in broiler chickens, and exposure to acute HS conditions can change their gene expression in the hypothalamus and digestive tract, respectively [17]. The hypothalamus is extremely vulnerable to environmental factors and orchestrates body energy balance and food intake [18]. The research objectives in this field are to combine existing scientific knowledge on these molecular mechanisms, to identify knowledge gaps, and to target practical goals for nutritional, genetic or managerial interventions to ensure food intake under stress. This will involve detailed knowledge of the role of hypothalamic neuropeptide signaling, inflammatory cytokines, oxidative stress sensors and the gut–brain axis. While many studies have explored the effects of one environmental stressor on chickens’ physiology and behavior, knowledge of the effect of many environmental stressors on molecular responses involved in control of feed intake is still quite limited. The objective of this review is to summarize the current knowledge about environmental stressors (e.g., thermal, nutritional, social, and management-related) and the neuroendocrine and hypothalamic mechanisms that affect appetite and feed intake in chickens. In addition, to discuss the molecular responses that control feed intake, and suggest potential molecular targets and management options to increase feed intake and make chickens more resilient when exposed to environmental stressors.

## 2. Literature Search Strategy

The information on the effects of environmental stress on feed intake in chickens was synthesized from structured, transparent, and reproducible studies, emphasizing the role of neuroendocrine regulation, appetite-related pathways in the hypothalamus and how this relates to epigenetic changes and mitigation strategies. To minimize selection bias and to get a broad range of literature pertaining to environmental stressors, appetite control, and nutritional or management interventions in poultry, a methodical search technique was employed.

### 2.1. Search Databases, Sources, Strategy, and Keywords

Two major electronic databases, Scopus and Web of Science, were used for searching the literature. The selected databases span the fields of agricultural, veterinary, physiological and molecular sciences and have a wide coverage. The reference lists of eligible full-text articles and articles that were included in the relevant review papers were also hand-searched (snowballing) to find other relevant studies missed by the electronic search.

The search strategy was designed using a combination of controlled vocabulary and free-text terms. The principal search terms were organized around the fundamental elements of the review: the species, the environmental stressors, the physiological target (feed intake and appetite), the regulatory mechanisms, and the mitigation strategies. Boolean operators (AND, OR) were employed to combine terms. A composite search string employed was: TS = ((chickens OR broiler OR layer OR poultry) AND (“environmental stress” OR “heat stress” OR “cold stress” OR “oxidative stress” OR “stocking density” OR “social stress” OR “immune stress” OR “LPS”) AND (“feed intake” OR appetite OR “food intake” OR “feeding behavior” OR “feeding behaviour” OR anorexia) AND (neuroendocrine OR neuropeptide OR “melanocortin” OR hypothalamus) AND (epigenetic* OR methylation OR “DNA methylation” OR “histone modification” OR “non-coding RNA”) AND (mitigation OR intervention OR supplement* OR “feed additive” OR management OR genetic selection OR “thermal conditioning”)) AND (PY = 2000–2025) AND (DT = Article) NOT (DT = Review OR DT = Proceedings Paper OR DT = Retracted Publication OR DT = Correction OR DT = Early Access OR DT = Editorial OR DT = Meeting Abstract). This Boolean string served only as an initial retrieval tool within a broader narrative-review methodology; it was supplemented by snowballing and targeted searches for relevant papers that did not satisfy all search conditions simultaneously.

### 2.2. Inclusion and Exclusion Criteria

The review focused on the effects of environmental stressors on feed intake in chickens and the underlying neuroendocrine, hypothalamic, and epigenetic mechanisms, as well as mitigation strategies, using the following criteria: Inclusion Criteria: Peer-reviewed original research articles (including in vitro, in vivo, and farm-based studies) published in English. Studies of environmental stressors (thermal, oxidative, social, nutritional, management-related) affecting feed intake, appetite control, neuroendocrine pathways, hypothalamic gene expression or epigenetic modification in chickens. Evaluation of mitigation measures (nutritional, management, genetic) affecting feed intake under stress. In order to reflect the contemporary period in poultry stress physiology and neuroendocrine research (2000–2025), with a limited number of more recent sources added for explanatory purposes only, articles were selected, and seminal older articles were included where they were relevant to the established mechanisms. Exclusion Criteria: Opinion articles, editorials, abstracts, and articles in languages other than English. Research on non-avian species that had no relevance to poultry (such as mammal or fish studies that were relevant only in general biological hypotheses) was included only where specifically identified and justified. Research conducted on interventions in which the specific impact of the environmental stressor or mitigation measure on feed intake was not apparent. Studies conducted with measures of production performance only (e.g., body weight gain, feed conversion ratio) and no measure of feed intake or appetite regulation.

### 2.3. Study Selection and Data Extraction

**Figure 1:**
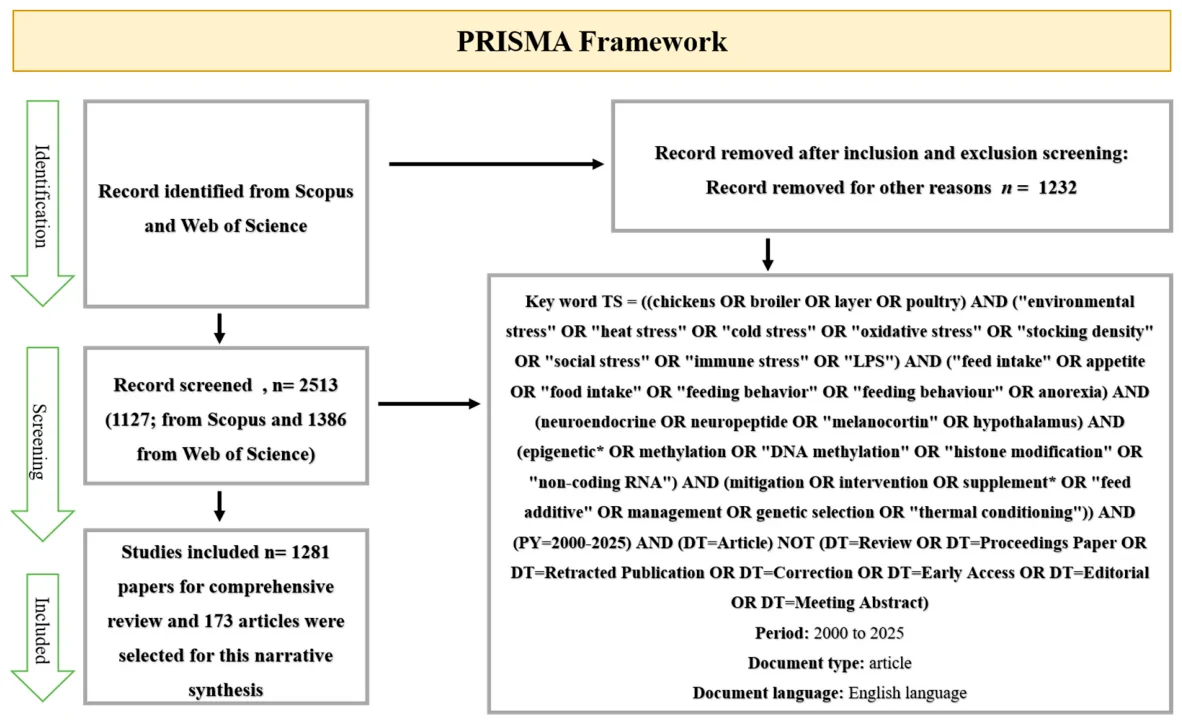
PRISMA framework for the review of Environmental Stress and Feed Intake in Chickens: Neuroendocrine Mechanisms and Mitigation Strategies.

The preliminary search conducted on 17 May 2026 resulted in 2513 articles from the two databases (Scopus: 1127; Web of Science: 1386). After discarding duplicate articles by authors using reference management software (EndNote, version 21), 1312 articles were left. Following the inclusion/exclusion criteria, the full texts of 1281 papers were selected for screening, based on title and abstract for comprehensive review, while 173 articles were chosen for this narrative synthesis (Figure 1).

The information gathered from these papers included the author, year of publication, experimental design, model system (broiler, layer, breeder, cell line), nature of the environmental stressor, dose/intensity of the stressor, duration of exposure, use of a mitigation strategy (if any), and main results relevant for the following themes: feed intake, neuroendocrine regulation, hypothalamic appetite pathways, epigenetic mechanisms, and mitigation strategies.

The approach for collecting the literature ensures that the narrative of this review is firmly grounded in a solid and clearly structured body of evidence, and that evidence directly demonstrated in chickens is separated from evidence used to support more general biological hypotheses.

## 3. Environmental Stressors Affecting Chickens’ Feed Intake

### 3.1. Thermal Stress

Stress induced by both heat and cold is a significant factor in the decrease of feed intake in chickens, due to physiological, hormonal and behavioral factors, and can cause significant economic losses in the world’s chicken production. Modern broiler chickens are a highly selected strain of chickens that are fast-growing and have high feed efficiency, which results in high metabolic heat production and thus are more susceptible to thermal stress [19]. The adverse effects of HS on laying hens affect their production performance and egg quality [20].

#### 3.1.1. Heat Stress (HS)

HS syndrome is defined as ambient temperatures that are above the chickens’ thermoneutral range; in broiler chickens, this means ambient temperatures exceeding 30 °C [18], although the actual threshold is strongly influenced by humidity, air speed, breed, age, and management practices. Accordingly, the Temperature–Humidity Index (THI) is commonly used to provide a more precise definition of heat stress conditions [21], which results in a cascade of negative impacts on chickens. HS, from a thermophysiological point of view, is defined as a disturbance of the normal thermoregulatory mechanisms which occurs when the thermal output from an animal’s metabolism becomes greater than the capacity for body heat to be lost to the environment [22]. Chickens are homeothermic endotherms with the ability to produce considerable body heat and use evolutionarily conserved mechanisms to maintain a relatively high and stable body temperature [23]. A notable result is a significant reduction in feed intake; this decrease represents a direct physiological response as the birds attempt to minimize metabolic heat production, thereby alleviating their internal heat load [18,24]. The hypothalamus plays a pivotal role in regulating food intake; during acute heat stress, the levels of hunger-stimulating neuropeptides within it, such as neuropeptide Y (NPY), often decrease, while satiety signals may increase [25]. Research indicates that HS induces changes in the neuroendocrine system, including alterations in the functions of endocrine glands such as the pituitary, adrenal, thyroid, and reproductive glands, which ultimately affect the rate of feed intake [24]. HS in chickens influences the levels of plasma leptin and ghrelin, hormones that regulate appetite [26]. The physiological factors responsible for reduced feed intake under HS are complex. HS causes a rise in body temperature, leading to metabolic changes and reduced weight gain. Broilers exposed to acute HS exhibit behavioral changes such as increased panting and wing spreading to dissipate heat, along with reduced activity resulting in decreased feed and water intake [27,28]. In broilers, chronic cyclic HS can cause a decrease in feed intake and growth rates from about day 29 to day 42 [27]. In the jejunum, a marked reduction in nutrient digestion and absorption is seen that is probably due to decreased levels of nutrient transporters and negatively impacts feed utilization efficiency and growth rates [29]. Water balance and intestinal integrity are another area of impact by HS, as are oxidative stress and inflammation factors that can help to diminish appetite and the efficiency of nutrient absorption [30]. It has been shown that glutamine can significantly reduce the damage to intestinal anatomical structure, inflammatory responses and intestinal barrier integrity due to heat stress, showing the importance of gut health in maintaining feed intake during HS [31]. Oxidative stress has a negative impact on broiler chickens’ welfare and productivity, and HS is a factor that aggravates the situation and, therefore, reduces feed intake.

#### 3.1.2. Cold Stress

The effect of cold stress on feed intake in chickens is bidirectional and depends on the severity and duration of exposure. Feed intake may initially be increased to generate extra metabolic heat to keep body temperature up and maintain feed intake during early growth stages in response to cold stress (for instance, exposure to 18 °C for 8 h daily during the first 14 days of a chick’s life) [32]. In contrast, chronic or severe cold stress, especially when energy supply is insufficient or combined with other stressors, can lead to reduced feed intake due to thermoregulatory limits and sustained hypothalamic–pituitary–adrenal (HPA) axis activation. The effects of daily cold stress (18 °C for 8 h) during the first week of broiler chick rearing on subsequent performance and physiological metabolism have been shown to be of a detrimental nature from day 1 to 35 [32]. Exposure to cold can disturb the body’s homeostasis, especially the gut health of young broiler chickens, due to its ability to change blood serum and intestinal barrier molecules’ chemical composition, energy metabolism and growth rates [33,34]. This dysfunction of gut health could manifest indirectly as a slowdown in feed intake due to inadequate absorption of nutrients and overall discomfort in the gut. In addition, studies in chickens have shown that cold stress can induce oxidative stress and impair intestinal integrity, which may negatively affect physiological status and feed intake [32,35].

HS or cold stress ultimately causes disruption of an organism’s delicate biological balance called homeostasis, which initiates a coordinated stress response. This includes redirecting energy and resources from growth and reproduction toward survival mechanisms and physiological changes (such as thermoregulatory adaptations in response to heat stress; thermoconservation via increased metabolism to combat cold stress), resulting in a negative impact on other functions such as digestion and nutrient absorption. These trade-offs may include a decrease in feed consumption and feed utilization efficiency.

### 3.2. Humidity and Ventilation

Humidity and ventilation are the most important environmental factors to consider and have a significant effect on feed intake rates and the overall performance of chickens, mainly because of their relationship with the birds’ thermoregulation and respiratory function. Relative humidity (RH) is a measure of how saturated a sample of air is with water vapor, such that a higher value would be associated with more water vapor per unit of volume in the air sample, and a 100% RH would indicate that the air sample is saturated with water vapor, which leads to condensation [36]. High relative humidity in the chicken house is detrimental because it makes it difficult for the birds to dissipate heat through evaporative cooling, as evaporative cooling depends on the evaporation of water from the respiratory tract; therefore, high humidity in the environment will reduce the moisture gradient between the bird’s body and the environment, making it harder for the birds to cool their bodies [11]. Thus, the bird’s internal temperature rises, making the effects of HS more severe [37]. Studies on White Pekin ducks have investigated the effect of different humidities on growth performance, slaughter characteristics and quality of the meat and concluded that optimal ambient humidity should be determined for optimal growth [38]. This accurate prediction and control of humidity would be helpful in resisting the incidence of disease in waterfowl.

Poor ventilation can be associated with increased concentrations of fine particulate matter (PM), ammonia (NH3), carbon dioxide (CO2) and other toxic gases within the chicken facilities that increases the effect of high humidity and temperature [39,40,41]. Birds are sensitive to ammonia levels above 25 ppm and use the following as criteria for the negative impacts: weight gain, feed conversion efficiency, survival rates and immune function. Humidity, ventilation rate, air speed and temperature are some of the factors that affect ammonia volatilization [40]. Increased levels of these pollutants can also lead to respiratory problems, reduced appetite, and oxidative stress, which further affects feed intake due to HS [30,40]. Therefore, proper control of humidity and ventilation inside the chicken house is crucial to reduce HS and minimize increases in harmful airborne contaminants in the air and to maximize feed intake which has a significant impact on the growth, welfare, and economics of chicken farming.

### 3.3. Social Stressors

Social stress factors, especially crowding and competition, have a significant influence on chickens’ feed intake, which can have a negative impact on chickens’ health, welfare and production. High stocking density (HSD) is a stressor that occurs very often in intensive chicken production. While HSD can increase production efficiency due to space utilization, it has also been shown to increase aggressive interactions, competition for resources, and stress in bird species [42,43]. The number of birds accommodated directly influences the welfare and performance of birds. There is some research to suggest that high stocking densities may lead to a significant decrease in average daily feed intake (ADFI) and hen-day egg production in laying hens [44]. In broilers, increased stocking density is associated with reduced growth performance and increased corticosterone (CORT) levels, which is an important index of stress. A study on broilers showed that 15 birds/m^2^ (high density) resulted in poorer performance and physiological responses than 7.5 birds/m^2^ (low density) [45]. The effect of crowding on feed intake is complicated. If kept at high densities, broilers might spend more time around the feeder without actually eating, meaning that eating behavior is limited because of competition or limited feeding space. The “restricted feeding behavior” directly follows from the crowding phenomenon, which is the physical proximity of the other birds which negatively affects an individual’s ability to obtain food efficiently [46]. This is similar to interference competition where one organism blocks another’s use of a shared resource. The negative effects of high stocking density stress include increased inflammatory responses, impaired immune system function and impaired redox balance, which all reduce feed intake and performance [42]. Furthermore, other stress factors such as higher stocking density may make animals more prone to infection, thus emphasizing the importance of understanding their impact on immune responses and how to manage stress.

Feeding competition is significant due to social rankings and limited food, and it really depresses feed consumption. Social animals, like hens, have a hierarchy of relationships, and they dictate access to resources (such as food) [47]. In some animals, vocalizations are observed as aggressive and influence feeding behavior. In chickens, the establishment of “pecking order” (aggression) is hormonally regulated (sex hormones) and can influence nutrient availability [48]. Other avians may be provoked to fight, particularly at feeding stations, thus affecting resource utilization [49]. Furthermore, the gut microbiota is crucial in influencing the connection between social stress and food consumption. Stress-related dysbiosis can increase gut permeability and change variables within the brain in the laying hen by affecting the microbiota–gut–brain axis, which can affect their negative behavior and their well-being [50]. Social stressors significantly reduce feed consumption, directly altering chickens’ behavior, inducing stress hormones like CORT, and affecting gut function and immunity. Beyond social stressors, other environmental and management-related factors also contribute to stress-induced reductions in feed intake. Seasonal stressors, particularly winter and other cold-related conditions, impose additional thermoregulatory demands that can reduce voluntary intake when energy supply is insufficient [35]. Feed quantity, as distinct from feed quality, is a further determinant: restricted availability or inconsistent supply directly limits intake and can induce competition and chronic stress [51,52]. Similarly, water availability is essential, as even short periods of restricted access can rapidly depress feed consumption and impair digestion and nutrient absorption. Understanding and addressing these stressors are crucial for improving the well-being of chickens and optimizing productivity in a commercial setting.

### 3.4. Light and Noise Pollution

Feed intake is an important growth parameter which is strongly affected by light and noise pollution, both abiotic stressors in modern chicken production, and therefore affects growth performance, welfare and productivity. The role of light as an environmental regulator also has significant impacts on the physiology of chickens, including their behavior, development, productivity and reproduction, as it synchronizes their 24 h rhythms and modulates neuroendocrine signals and metabolic pathways [53]. These elements are the intensity, the duration (photoperiod), and the spectrum of the light. Growth performance of broilers is reported to be affected by low light intensity (<5 lux) [54]. On the other hand, over-exposure or incorrect exposure to light can disrupt circadian rhythms, increase blood levels of stress hormones such as CORT, and alter the release of melatonin, and all of these can lead to reduced appetite and food consumption as shown in quail [55]. The effect of artificial photoperiods on feed intake has been observed as negative in broiler chickens, in which continuous light (24L) resulted in a lower ADFI than intermittent light (4L:2D) [56]. Japanese quail fed at a medium light intensity (50 lux) consumed more feed than did those fed at a low (10 lux) or high (250 lux) intensity [57]. Other illumination of feeder lines has been observed to influence broiler distribution and feeding behavior, with a gradient of feeder illumination (100 lux) and pen illumination (4 lux) as shown in turkeys [58]. Furthermore, the use of light pulsing from LED luminaires, particularly frequencies less than 488 Hz, can also reduce livability and feed efficiency of layers, as shown in geese [59]. Flickering LED lights could be a stressor to male fattening turkeys, influencing their behavior and welfare [60]. Different wavelengths of light have effects on broiler behavior—influencing activity and/or their interaction with feed. For example, green light has been shown to reduce oxidative stress and inflammation in geese due to heat stress, indirectly improving feed intake, which means better overall health [61]. The ability of chickens to detect ultraviolet (UV) light suggests that UV light may improve their well-being and optimize production by providing a more natural environment, which could also have a beneficial effect on their feed intake by increasing the vitamin D pathways in the body [62]. Precision feeding systems for broiler breeders, where continuous access to feed is required and supplemental feed is necessary throughout the day, can potentially have an effect on growth and reproductive performance; therefore, careful assessment of lighting systems is important.

**Figure 2:**
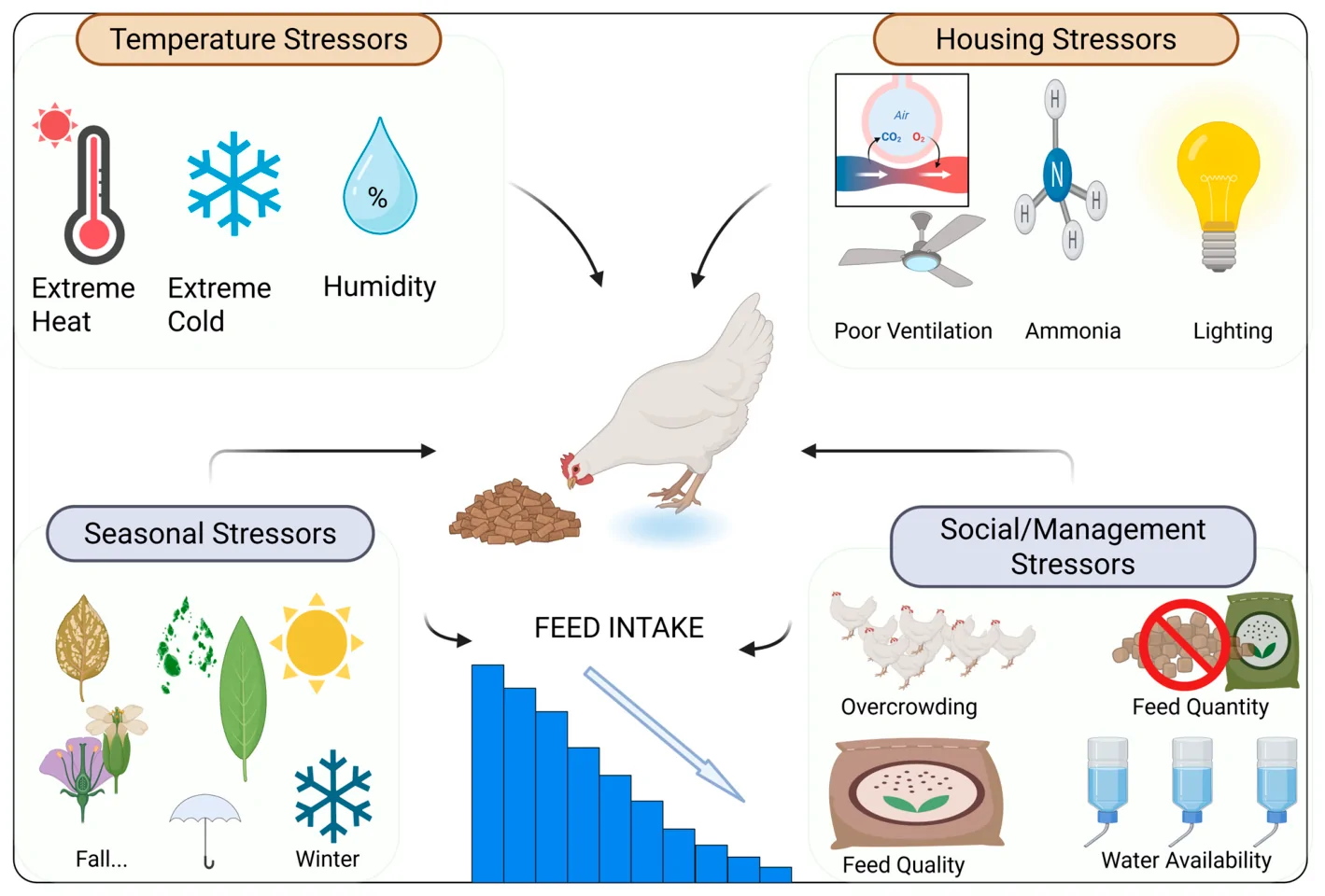
This is a general indication of the major stress factors that impact the feed intake of chickens, such as temperature (heat, cold, humidity), housing (ventilation, ammonia, lighting), seasonal change, and socio-administrative factors (overcrowding, feed quality, water). Created in https://BioRender.com. Accessed on 11 October 2026.

Noise pollution, a notable abiotic stressor, significantly impacts chickens’ feed consumption. Chronic noise, particularly at levels exceeding 85 dB or rapid, unpredictable sounds (e.g., machinery, human activity), elicits acute stress responses through the HPA axis, resulting in diminished eating drive, heightened vigilance, and gastrointestinal suppression [63,64,65]. Prolonged exposure to high-decibel noise (85–95 dB) in pullets has been demonstrated to generate stress, impacting their behavior, CORT levels, and intestinal barrier function [66,67]. Noise levels in laying hen houses may be high due to automation and large flock sizes, which need to be assessed and measures taken to reduce them. This sensory pollution will upset biological processes, from cellular mechanisms to entire ecosystems. Figure 2 classifies the main stressors which impact chickens’ feed intake.

## 4. Physiological and Behavioral Responses to Environmental Stressors

### 4.1. Changes in Feeding Behavior

Complex physiological responses in birds are induced by environmental stressors affecting feed intake and bird health, such as high and low temperatures, light spectrum variations, noise and overcrowding. Hematologic stress in chickens can lead to pale, soft, exudative (PSE) meat in short-term transportation and dark, firm and dry meat (DFD) in long-term transportation and seasonal changes, which negatively affects meat quality [68]. Prolonged exposure to heat in laying hens can lead to kidney damage, possibly through the production of a uremic toxin, indoxyl sulfate (IS) [69]. The cellular-level effect of HS is excess production of reactive oxygen species (ROS), which causes oxidative stress. The HPA axis is a major physiological pathway in response to stress and results in elevated CORT [70,71]. This neuroendocrine pathway is an important way to respond to stressors, but it can cause an enhanced adaptive load or an increase in stress load on body systems if used continuously. The heterophil-to-lymphocyte (H/L) ratio is a well-established stress marker in chickens which reflects the physiological response to negative environmental factors such as heat stress, cold stress, water deprivation, etc [15]. Anecdotally reported, but not often studied, is the phenomenon of feather sucking or licking in meat chicken breeder birds, which may have welfare implications [72]. Behaviors associated with piling are a significant issue, resulting in injuries, suffocation and greater stress in non-cage laying hens, which negatively affects immune function and susceptibility to disease [73]. The mechanical loading of broiler chickens in transportation may lead to escaping, flapping and collisions as signs of stress and animal welfare issues [74]. New technologies such as the integration of improved Cascade Region-Based Convolutional Neural Networks (Cascade R-CNN) have been developed to detect and monitor the HS behavior of broiler chickens with yellow-feathered appearance to enable quick interventions to minimize losses [75]. When birds are stressed, they tend to be more aware and have longer feeding periods between meals. As temperature increases, feeding activities can be moved to cooler parts of the day (e.g., early morning or late evening) as a behavioral adaptation. Normal foraging and competitive feeding patterns can be further disrupted by stereotypical responses and social isolation arising from chronic stress.

### 4.2. Alterations in Metabolic Rate

Chickens, particularly commercial broiler chickens, have a higher metabolic rate, which results in greater production of internal heat and increases their vulnerability to heat stress. Acute stress typically increases metabolic rate, leading to increased oxygen consumption and energy expenditure, mostly via the activation of the sympathetic nervous system and release of the catecholamines. In contrast, chronic stress may lead to inhibition or disruption of metabolism, such as reduced feed consumption, inadequate mitochondrial efficiency and a switch to catabolic metabolism, including gluconeogenesis and muscle protein degradation [76]. Studies have shown that broiler chickens affected by HS consume less feed and gain less body weight with an increase in feed conversion ratio [77,78,79]. When laying hens experience HS, they experience reduced egg production, quality and metabolic rate [80]. Heat shock proteins (HSPs) are important components of the cell’s HS defense system. Such evolutionarily conserved proteins function as molecular chaperones and are involved in the correct folding of proteins and in the protection of the cell from damage. Chickens are known to increase their levels of heat shock proteins, in this instance, HSP70, as a response to heat stress, as a way of keeping the cell in homeostasis [81]. This metabolic change is reflected in altered hormones in metabolism. HS can cause a decrease in thyroid hormones (T3/T4) that are necessary for metabolic rate regulation, and also stimulates the HPA axis in the hypothalamus, increasing CORT levels. High levels of CORT and other stress hormones, such as adrenaline, through the sympatho-adreno-medullary (SAM) nervous system shift energy away from growth or production and toward short-term survival reactions [82]. Liver dysfunction due to oxidative stress may have a negative effect on lipid metabolism, which is crucial for energy supply and egg production in birds [83]. In addition, HS can lead to lung injury through the following mechanisms: weakening the blood–air-pulmonary barrier and activation of inflammatory signaling pathways that affect both lung function and metabolic efficiency.

The digestive system is especially vulnerable to HS, which alters the gut microbiota, intestinal architecture and expression of tight-junction proteins, causing greater intestinal permeability or “leaky gut syndrome” and systemic inflammation. These alterations worsen the metabolic disturbances and nutrient malabsorption [84,85]. The effect of cold-induced stress on metabolic rate is also different. Birds generate metabolic heat at low temperatures to maintain a stable body temperature, particularly if they cannot use behavioral thermoregulation. This increased metabolism may lead to increased feed consumption and feed conversion ratio. The research shows that low temperatures cause a significant rise in live body weight, food consumption and feed conversion ratio in laying hens, which means that low temperature increases the need for heat generation [86]. Sympathetic nervous system activation and increased levels of uncoupling proteins can increase basal metabolic rate (BMR). Stressors such as management, disease threats, and feed deficiencies can set in a chain of hormonal reactions that compromise immunity and cause disturbance of the metabolism [87]. This may include decreased feed intake, lethargy, feed conversion inefficiency and poor weight gain.

### 4.3. Stress-Induced Hormonal Changes

These stresses can trigger a chain of physiological responses, mainly hormone changes, that can have a profound effect on chickens’ health, well-being and productivity. In chickens, the major hormonal response to stress is activation of the HPA axis, which leads to an increase in CORT, the major avian glucocorticoid [80]. This neuroendocrine system is an important adaptive response, but it can have significant adverse effects when it is continually stimulated. HS activates the HPA system, which in turn causes the release of corticotropin-releasing factor (CRF) from the hypothalamus. This, in turn, stimulates the pituitary gland to release adrenocorticotropic hormone (ACTH) that acts on the adrenal cortex to increase the level of CORT in the blood [24]. A study evaluating the effect of a concentrated yeast fermentation product on broiler chickens suffering from feed interruption and HS showed that the stress factor significantly increased plasma CORT levels [70]. Chronic stress, caused by environmental conditions such as extreme temperatures, malnutrition, or high animal density, leads to a state of “adaptive overload” or physiological deterioration. This results in dysfunction of the HPA axis, leading to impaired growth, reduced reproductive efficiency, and metabolic changes.

### 4.4. Impact on Growth and Productivity

Research indicates that broiler chickens exposed to HS exhibit less body weight gain compared to chickens raised in moderate-temperature environments. A study conducted on broiler chickens aged 17 to 31 days revealed that heat stress, assessed using the temperature–humidity index (THI), resulted in reduced body weight [88]. A separate study comparing industrial broiler chickens with domestic hybrid chickens revealed that body weights decreased under summer HS conditions (average temperature 34.41 ± 0.52 °C) in contrast to control conditions (average temperature 23.68 ± 0.39 °C) [89]. Poor growth is exacerbated by poor nutrient absorption and reduced intestinal integrity resulting from oxidative stress and inflammation caused by heat stress.

In addition to growth, broiler chickens suffering from HS often experience a decrease in carcass weight, dressing percentage, and breast meat percentage. Chronic cyclical HS has been shown to reduce breast meat percentage and increase abdominal fat percentage in broiler chickens [88]. Reduced muscle mass and changes in fat distribution negatively impact the overall quality and economic value of chicken products. Feed withdrawal, sometimes used as a management approach, has been shown to affect the growth performance and carcass characteristics of broiler chickens subjected to heat stress, depending on the timing of the feed withdrawal [90]. Additional environmental stressors, such as high storage density and unsuitable housing conditions, may exacerbate the effects of heat stress, leading to reduced development and production [91]. Chronic HS in laying hens leads to decreased egg production, deterioration of egg quality (such as shell thinning and reduced white height), and affects blood biochemical parameters and uterine gene expression [92,93]. Certain nutrients, such as the arginine/lysine ratio, can affect performance, egg quality, and immune parameters during cyclical HS conditions [94]. This requires the formulation and implementation of effective mitigation techniques to ensure sustainable chicken production.

## 5. Molecular Mechanisms Underlying Stress Responses

### 5.1. Stress Signal Transduction Pathways

The HPA axis is a key component of the avian stress response. In response to stress, corticotropin-releasing hormone (CRH) is released from the hypothalamus, which stimulates the anterior pituitary gland to release ACTH [95]. ACTH hormone subsequently affects the adrenal gland, facilitating the synthesis and secretion of glucocorticoids, especially CORT in birds [96]. CORT is essential for regulating metabolism, immune response, and behavior to enable adaptation to stress. Research indicates that exogenous CORT treatment effectively induces a stress model in chickens, mimicking physiological stress responses such as modulation of immune function and gene expression in various tissues, including the spleen and thymus [97,98]. HPI axis function depends not only on CORT levels, but also on the presence of free active CORT and its enzymatic activation by 11β-hydroxysteroid dehydrogenase (11β-HSD) [96]. Glucocorticoid receptors (GRs) facilitate the effects of CORT by functioning as ligand-activated transcription factors. Upon binding to CORT, GRs translocate to the nucleus and modulate the expression of stress-responsive genes. Differential expression of GRs has been noted in several brain areas implicated in stress regulation, including the nucleus of the hippocampal commissure and the paraventricular nucleus in chickens [95]. The effects of glucocorticoids are specific, depending on factors such as the bioavailability of the hormones, the differing affinities of the receptors, and slow genomic and rapid non-genomic signaling pathways.

The nuclear factor kappa-light-chain-enhancer of activated B cells (NF-κB) pathway is a key regulator of inflammatory and immune responses to a variety of stresses such as oxidative stress, thermal stress and pathogen exposure. In chickens, persistent stress may result in systemic inflammation and oxidative stress, frequently associated with NF-κB activation. Intermittent mild cold acclimation has been demonstrated to alleviate intestinal inflammation and immunological dysfunction in cold-stressed broilers via modulating the toll-like receptor 4 (TLR4)/MyD88/NF-κB pathway [99]. The NF-κB pathway activation often entails the proteasomal degradation of its inhibitor, IκB, facilitating the translocation of NF-κB to the nucleus to start gene transcription [100]. Disruption of NF-κB can play a role in the development of chronic inflammatory diseases.

The mitogen-activated protein kinase (MAPK) cascades are crucial signal transduction pathways that transduce multiple extracellular signals to specific intracellular targets and responses, including stress response, proliferation, differentiation, and inflammation. In chickens, stressors such as HS and oxidative stress activate MAPK pathways such as p38, c-Jun N-terminal kinase (JNK) and ERK. In black-boned hens, resveratrol has been proven to reduce HS-induced damage to the jejunal mucosa through its regulation of the TLR4/MAPK signaling pathway. The MAPK cascade is a hierarchical system of kinases (MAPKKK → MAPKK → MAPK) that is used to amplify signals and thus produce a strong cell response to a low level of initial cell stimulation. Various MAPK pathways can elicit divergent cellular responses; for example, JNK and p38 MAPK are frequently correlated with stress responses, but ERK pathways are generally connected with growth and proliferation [101]. Many stressors in chickens, such as thermal stress, transportation stress, and immunological challenges, result in heightened production of ROS and reactive nitrogen species (RNS), thus inducing oxidative stress. Oxidative stress can harm cellular components, undermine intestinal integrity (resulting in “leaky gut syndrome”), and impair immunological function [102].

### 5.2. Gene Expression Changes

Environmental stresses, especially HS, cause substantial changes in hypothalamus gene expression that directly influence feed intake in chickens. The principal hypothalamic appetite-regulating genes influenced by environmental stress encompass the orexigenic neuropeptides NPY (neuropeptide Y) and AgRP (agouti-related peptide), as well as the anorexigenic neuropeptides pro-opiomelanocortin (POMC) and CART (cocaine and amphetamine regulated transcript). The peripheral lipopolysaccharide (LPS) challenge, which simulates acute immunological stress, markedly reduced hypothalamic mRNA levels of NPY and AgRP while elevating anorexigenic POMC expression in broilers [103,104]. Transcriptome study of hypothalamic responses indicates that POMC serves as a central gene, interacting with the highest number of nodes and ingestion-related pathways, including associations with CRH, AgRP, NPY, thyrotropin-releasing hormone (TRH), vasoactive intestinal peptide (VIP), and thyroid-stimulating hormone beta subunit (TSHB) [105]. Cyclic HS studies comparing unselected junglefowl with modern broiler lines (slow-, moderate-, and fast-growing) revealed that the expression of key feeding-related hypothalamic neuropeptides (NPY, AgRP, POMC, CART) remained predominantly stable following prolonged exposure. Melanocortin receptor 1 demonstrated a line-dependent decline from junglefowl to contemporary broilers, although visfatin mRNA levels were higher in junglefowl compared to selected lines under thermoneutral conditions [25]. The findings indicate that adaptation to prolonged heat exposure involves predominantly stable hypothalamic circuits; however, the line-dependent decline in melanocortin receptor 1 suggests that genetic selection does influence specific components of the hypothalamic melanocortin system. The hypothalamic AMP-activated protein kinase (AMPK) pathway regulates feed intake during temperature stress by altering the production of NPY and POMC. N-acetyl-L-cysteine supplementation has been studied as a potential intervention influencing hypothalamic AMPK-related gene expression during chronic HS in laying hens [106]. Table 1 summarizes the gene expression alterations mediated by stressors on feed intake in chickens.

### 5.3. Neuroendocrine Factors Influencing Feed Intake

Chicken feed intake is regulated by a sophisticated neuroendocrine system where peripheral hormones interact with central hypothalamic circuits to sustain energy equilibrium. Key hormonal factors, such as ghrelin, leptin, CORT and other neuropeptides in the hypothalamus, form a coherent signaling system that responds acutely to environmental disturbances.

Ghrelin is an endogenous ligand of the growth hormone secretagogue receptor (GHS-R), primarily synthesized in the glandular stomach of chickens. However, in contrast to mammals, where ghrelin is predominantly orexigenic, ghrelin in chickens generally exerts anorexigenic effects, suppressing feed intake following central or peripheral administration. This species difference appears to involve the CRF system, which mediates ghrelin-induced anorexia in neonatal chicks [115]. Moreover, chicken ghrelin was first purified and characterized from the proventriculus (glandular stomach), and its structure and tissue distribution differ from those of mammalian ghrelin [116]. These findings highlight that the ghrelin system in chickens cannot be directly extrapolated from mammalian models and should be interpreted within an avian-specific neuroendocrine framework [117].

It has been identified in brain tissue, where neurons that immunologically react to ghrelin undergo developmental changes, and its presence in the embryo suggests an early role in growth and development [118]. Researchers studied acetylated ghrelin (AG) and des-acylated ghrelin (DAG) at different pulse frequencies and doses. They concluded that pulse frequencies and doses of AG and DAG had varying effects on feed intake (FI), average daily weight gain (ADG), CORT levels, and growth hormone (GH) in broiler chickens [119]. A crucial mechanistic understanding is that peripheral ghrelin suppresses food consumption via a HPA axis-dependent mechanism in chickens. One possible explanation is that ghrelin enhances the central activity of CRF, which in turn stimulates the HPA axis to increase CORT production in the adrenal gland [120]. It has been shown that the inhibitory effect of ghrelin on food intake is mediated by the corticotropin-releasing factor system in newborn chicks [121]. Lei et al. [17] examined the effect of acute HS (35 °C, 6 h, 65% relative humidity) on the gene expression of appetite-regulating peptides in 42-day-old broiler chicks. The results showed that the levels of ghrelin mRNA in the hypothalamus showed no statistically significant change (*p* > 0.05) following exposure to acute heat stress. However, a marked increase in ghrelin mRNA concentrations was observed in the gastric glands, duodenum, and jejunum. This suggests that ghrelin in peripheral gastrointestinal tissues, rather than in the hypothalamus, may be the primary regulatory target for acute heat stress-induced anorexia.

In contrast to mammals, where leptin is primarily produced by adipose tissue, leptin in chickens is predominantly expressed in the liver, with additional expression in adipose tissue and other organs [122]. Furthermore, the effects of leptin on feed intake and energy balance in chickens are less consistent than in mammals and appear to depend on dose, route of administration, and physiological state [123,124]. These differences indicate that the classical mammalian leptin–POMC–NPY/AgRP framework should be applied to chickens with caution and that avian-specific regulatory mechanisms must be considered. The hormones leptin and ghrelin significantly affect energy balance in chickens. They act as mediators for managing long-term energy balance and suppressing feed consumption [118]. In the hypothalamic feeding circuit, leptin stimulates appetite-suppressive POMC neurons and inhibits appetite-stimulating NPY/AgRP neurons, thereby reducing appetite [125]. Dysregulation of adipokine signaling, including central leptin resistance, is a key mechanism in metabolic diseases and is influenced by receptor affinity, homology diversity, and intracellular negative feedback loops [126,127]. The presence of both leptin and ghrelin in the embryo suggests their role in growth and development. The role of the leptin system in chickens in regulating various physiological activities is still under investigation, partly due to the controversial history of leptin gene identification in chickens and the contrasting tissue expression patterns compared to mammalian models [118].

**Figure 3:**
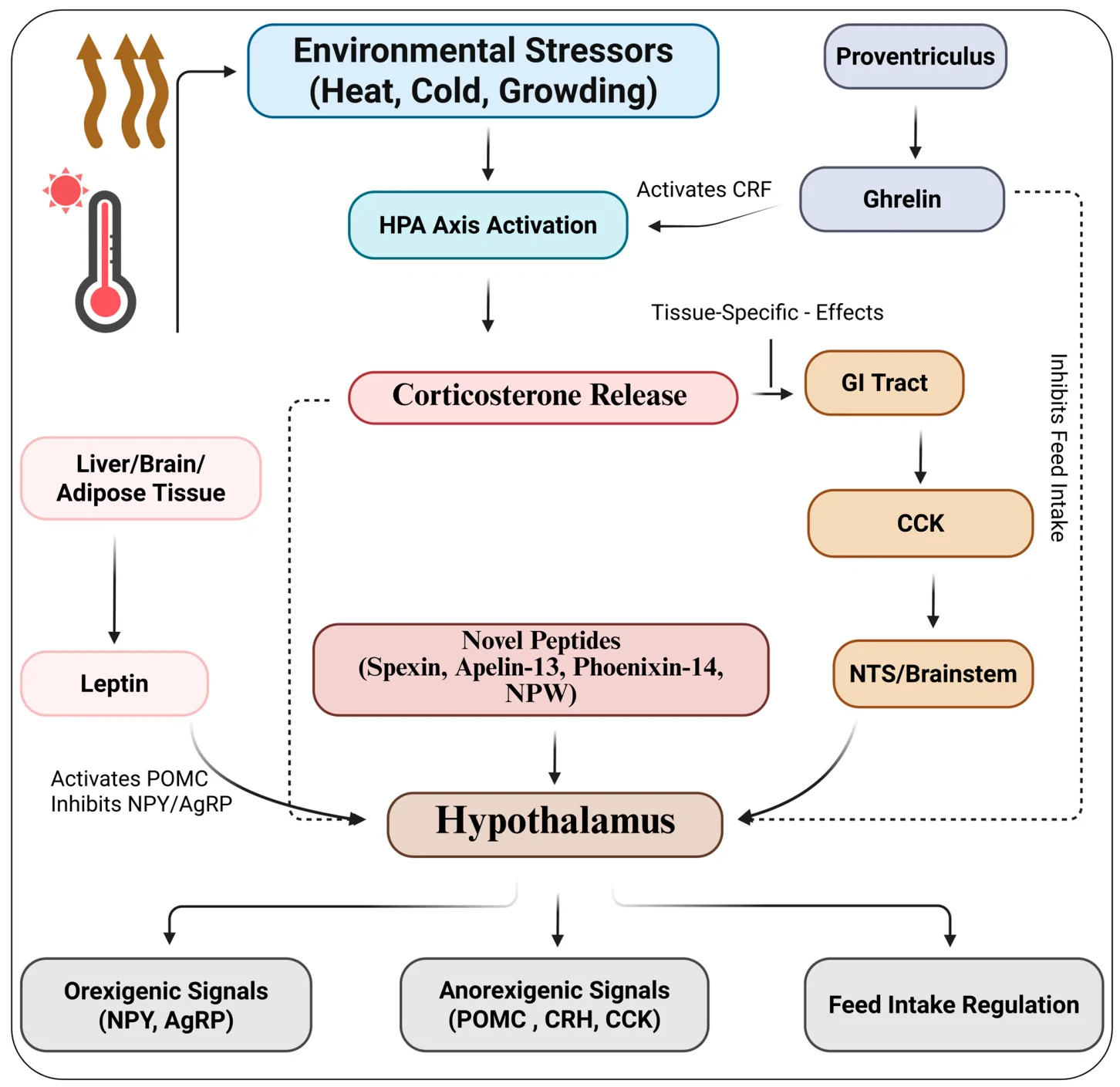
Environmental stressors, including heat, cold, and crowding, modulate feed intake in chickens through interacting central and peripheral pathways. Stressors activate the hypothalamic–pituitary–adrenal (HPA) axis, resulting in corticosterone release, which has tissue-specific effects on the gastrointestinal (GI) tract and central nervous system. Ghrelin, which is mostly generated in the proventriculus, suppresses feed intake and triggers CRF signaling. Peripheral satiety signals from the GI tract, including CCK, act on the nucleus tractus solitarius (NTS)/brainstem and are relayed to the hypothalamus. Leptin, produced by the liver, brain and adipose tissue, activates POMC neurons and inhibits NPY/AgRP neurons. In chickens, leptin’s effects on feed intake and energy balance are less consistent than in mammals and depend on dose, route of administration, and physiological state, so this framework should be applied with caution. Novel peptides, including spexin, apelin-13, phoenixin-14, and neuropeptide W (NPW), also converge on the hypothalamus. These signals are incorporated into the hypothalamus’s orexigenic (NPY, AgRP) and anorexigenic (POMC, CRH, CCK) outputs, which in turn control feed intake. Environmental stressors generally affect feed intake through interactions between appetite stimulators and suppressors in the peripheral and central nervous systems rather than through a single straight pathway. Created in https://BioRender.com. Accessed on 11 October 2026.

Liu et al. [110] performed a significant study: 48 laying hens were subcutaneously injected with CORT at a dose of 2 mg/kg of body weight or received a sham treatment. CORT administration resulted in a marked increase in hypothalamic mRNA levels of NPY, CRH, melanocortin 4 and 5 receptors (MC4R and MC5R), and cholecystokinin (CCK). Conversely, expression levels of POMC, AgRP, and melanocortin 1 receptor (MC1R) were significantly reduced. The data indicate that corticosterone simultaneously activates appetite-stimulating pathways (such as NPY) and appetite-inhibiting pathways (such as CRH and CCK), creating a complex regulatory environment. However, CORT administration led to a significant reduction in CCK mRNA levels in the jejunum and ileum, as well as in local ghrelin mRNA expression in the ileum (*p* < 0.05) [110]. This indicates that a dynamic balance between appetite-related peptides in the hypothalamus and the gastrointestinal tract determines the nutritional status of laying hens exposed to CORT. Reward signals, including leptin and ghrelin, have effects on food intake, which regulates energy balance. CORT also affects the configuration of the input structure of POMC and NPY/AgRP neurons. The relationship between stress and metabolism is vital to the understanding of the pathogenesis of metabolic diseases and stress-related disorders in the chicken production system. Table 2 shows the most important neuroendocrine mechanisms regulating feed consumption in birds, and Figure 3 shows that environmental stressors have multiple hormonal and neurohormonal effects rather than a single linear effect.

### 5.4. Non-Invasive Biomarkers of Stress-Induced Feed Reduction

One major need in poultry research is the lack of proven non-invasive biomarkers that can predict stress-induced feed reduction prior to productivity losses. CORT and its metabolites are among the most widely proposed candidates; nevertheless, there is currently little evidence to support their predictive value, which calls for a critical assessment. Because feather CORT builds up over time and can be measured without frequent handling, it has been extensively studied as a non-invasive biomarker of chronic stress in birds [133]. However, the association between feather CORT and acute or short-term changes in feed intake is still unclear, and it mostly represents cumulative glucocorticoid exposure over the course of feather growth rather than current stress condition [134]. Furthermore, threshold values for forecasting feed reduction have not been established, and factors including feather type, molt timing, and individual variance complicate interpretation.

Other non-invasive matrices have also been explored. Fecal CORT metabolites offer a non-invasive alternative to blood sampling, but their concentrations are influenced by diet, gut transit time, and metabolism, and they primarily reflect recent rather than cumulative stress [135]. Egg-based CORT has been proposed as a biomarker of stress in laying hens, as glucocorticoids are deposited into the yolk during follicular development; however, its interpretation is complicated by the long deposition window and the influence of hen age and laying rate [136]. Thermal imaging and panting/computer vision enable remote assessment of physiological and behavioral indicators of stress, such as surface temperature and respiratory rate, but these approaches remain sensitive to environmental conditions and require further validation under commercial settings [137].

The H/L ratio has also been suggested as a non-invasive measure of stress in chickens. Although the H/L ratio is a well-known stress indicator [138], it is usually determined using blood samples, which are intrusive and unsuitable for field monitoring in real time. Non-invasive alternatives, such as image-based or automated cell-counting techniques, remain in their infancy and require validation in commercial settings [139]. Furthermore, the H/L ratio’s predictive ability is limited since it simultaneously reflects immunological and stress status rather than forecasting future reductions in feed intake. Vocalization-based and wearable monitoring technologies have become viable instruments for assessing the well-being and behavior of chickens in real time. Changes in behavior, activity, and vocalization patterns that may precede or coincide with stress-induced reductions in feed intake can be detected by accelerometers, activity sensors, and acoustic monitoring devices [140]. These methods are still mostly experimental, though. Predictive attempts such as growth prediction in broilers [141] exist, but there is no systematic validation of their capacity to forecast feed reduction before it happens.

However, wearable/vocalization-based monitoring, feather CORT, and non-invasive H/L samples are significant steps toward non-invasive stress monitoring; yet, none of these methods has been proven to be predictive biomarkers of stress-induced feed decrease in chickens. In order to ascertain the viability and predicted accuracy of these methods, future research should concentrate on setting thresholds, standardizing sampling and analytical procedures, and evaluating them in real-world settings. Claims of non-invasive, real-time prediction of stress-induced feed decrease should be viewed as research potential rather than proven capabilities until such validation is accomplished.

## 6. Epigenetic Modifications Under Stress

The word “epigenetic” refers to changes to the genome that may be reversible and heritable without affecting deoxyribonucleic acid (DNA) sequences. These changes enable the single genome of multicellular organisms to produce several cell/tissue types or alter its transcriptional repertoire in response to the ever-changing environmental conditions [9,142,143,144]. In chickens, epigenetic modifications that affect heritable changes in the regulation of feed intake have emerged as essential molecular mechanisms driving persistent effects of environmental stressors on feed intake regulation [9]. Epigenetic changes, unlike rapid and short-term changes triggered by stress hormones and neurological pathways, can trigger lasting changes in gene expression that persist long after the stressor has passed, in some cases across generations. DNA methylation, post-translational modifications of histone proteins and non-coding RNA, in particular microRNAs (miRNAs) and RNA methylation (epitranscriptomics), are important epigenetic mechanisms involved in anorexia induced by stress [145]. These processes mediate the remodeling of feeding circuits at the chromatin level in birds when they are exposed to environmental stressors, and offer a biological substrate for anorexia associated with chronic stress, as well as targets for nutritional and pharmacological manipulation.

### 6.1. DNA Methylation: Transcriptional Silencing of Appetite-Regulating Genes

In chickens, there is increasing evidence that hypermethylation of promoter regions in important genes that control growth and feed intake is responsible for down-regulation of gene expression and continued loss of appetite because of HS and nutritional deficiencies. A good example of this phenomenon is research into embryonic thermal manipulation (TM), which involved exposing broiler eggs to higher temperatures (39 °C with 65% humidity from day 10 to day 18 of incubation), which led to chicks having significantly lower body weight, weight gain rates and average daily feed intake than the controls [146]. The mechanistic changes at the DNA level were related to hypermethylation of the promoter regions of the genes involved in insulin-like growth factor 1 (IGF1) and growth hormone receptor (GHR) in the liver, associated with their decreased expression [146]. The promoter region of the IGF1 signaling pathway showed hypermethylation, which is associated with altered hepatic gene expression, growth, and voluntary feed intake (VFI). Because this pathway links nutrition with growth and appetite, these epigenetic changes may contribute to the early-life HS-associated reduction in VFI, although a direct causal mechanism has not been demonstrated. Likewise, low-energy, high-fiber diets (nutritional stress) were associated with DNA methylation changes in both blood and liver tissue of laying hens. Although the number of differentially methylated CpG sites (DMCs) was limited, they were associated with genes playing a role in metabolic adaptation [147,148]. Fascinatingly, the number of DMCs that correlated with DEGs was limited, indicating that DNA methylation changes, under some circumstances, may be tissue-specific, or that other epigenetic mechanisms (such as histone modifications and microRNAs) may affect the transcriptional response to nutrition restriction [9]. The discovery of multiple epigenetic mechanisms involved in stress adaptation is a good illustration of the importance of multiple layers of epigenetic control.

### 6.2. Histone Modifications: Dynamic Chromatin Remodeling in Hypothalamic Feeding Circuits

Post-translational modifications (PTMs) of histones (HPTMs) affect the structure of chromatin and the accessibility of transcription factors without changing the DNA sequence. By contrast, the methylation of DNA is relatively stable, whereas many histone modifications are highly dynamic and enable the rapid and reversible changes in transcription in response to environmental stimuli [145]. In chickens, some of the HPTM molecules have been characterized as important regulators of anorexia in the hypothalamus and adrenal gland.

A pivotal study conducted on three-day-old chicks revealed that fasting-induced stress led to a marked increase in the dimethylation and trimethylation of histone H3 at lysine 27 (H3K27me2/me3) in the preoptic/anterior hypothalamus (PO/AH). This repressive histone modification, catalyzed by the methyltransferase EZH2 (Enhancer of Zeste Homolog 2), is associated with reduced gene expression of CRH in the paraventricular nucleus (PVN), a key mediator of appetite suppression. Unexpectedly, fasting enhanced the production of TRH, suggesting that H3K27 methylation may differentially regulate distinct sets of neuropeptides to coordinate adaptive metabolic responses to food deprivation [149]. These results highlight the potential significance of H3K27 histone methylation in responses to nutritional stress during early life and suggest the existence of an epigenetic pathway for subsequent adaptation to diet-induced stress. Under acute heat stress, rural laying hens exhibited distinct patterns of HPTMs in the adrenal gland, depending on their heat-tolerance phenotype; heat-sensitive birds showed a marked rise in body temperature and extremely high levels of the H3K27me3 marker in the adrenal gland, alongside positive interactions between this marker and adjacent modifications (such as K36me and K37me) [150]. While this study did not assess feed intake, these adrenal histone modifications may represent a mechanism contributing to the stress response, and their potential link to appetite regulation warrants further investigation. In contrast, heat-tolerant birds exhibited reduced levels of H3K9me, a repressive marker associated with heterochromatin formation. Diverse HPTM patterns are linked to adrenal glucocorticoid production and may influence genetic variation in heat tolerance. Adrenal H3K27me3 levels were directly correlated with the function of the HPA axis, suggesting that histone modifications in stress-responsive peripheral tissues contribute to systemic adaptation to stress and, indirectly, to stress-induced appetite suppression.

### 6.3. Non-Coding RNAs: MicroRNAs as Circulating Markers of Stress-Induced Anorexia

MicroRNAs (miRNAs) are short non-coding RNAs that post-transcriptionally regulate gene expression by binding to target mRNA, and are now identified as key regulators of stress response and appetite regulation in chickens. miRNA molecules can be shed into the blood and may be ideal minimally invasive biomarkers for quantifying stress and feed intake in vivo [151]. However, it should be noted that, although circulating miRNAs are considered minimally invasive, they still require blood sampling and therefore do not meet the criteria for fully non-invasive biomarkers. In a key study looking at the effect of feed deprivation in chickens, those with the highest and lowest residual feed intake (RFI) (high R+ and low R-) were found to have a different plasma “miRNome”. After 16 h of food deprivation, 23 miRNA molecules were found to be differentially abundant in the fed versus fasted condition, and 19 more differed between the two genetic lines. Some that stand out are gga-miR-204, gga-miR-let-7f-5p and gga-miR-122-5p, which are known to play regulatory functions in energy metabolism. In the context of feed deprivation-induced animal physiological changes, plasma miRNA molecules sensitive to stress responded rapidly to the changes, and this response was strongly affected by genetic background [151]. These results indicate that even slight differences in a gene can significantly affect epigenetic stress responses and could imply that miRNAs are promising candidates as markers to identify stress-induced loss of appetite in the industrial production of chickens.

### 6.4. RNA Methylation (Epitranscriptomics): A New Frontier in Appetite Regulation

**Figure 4:**
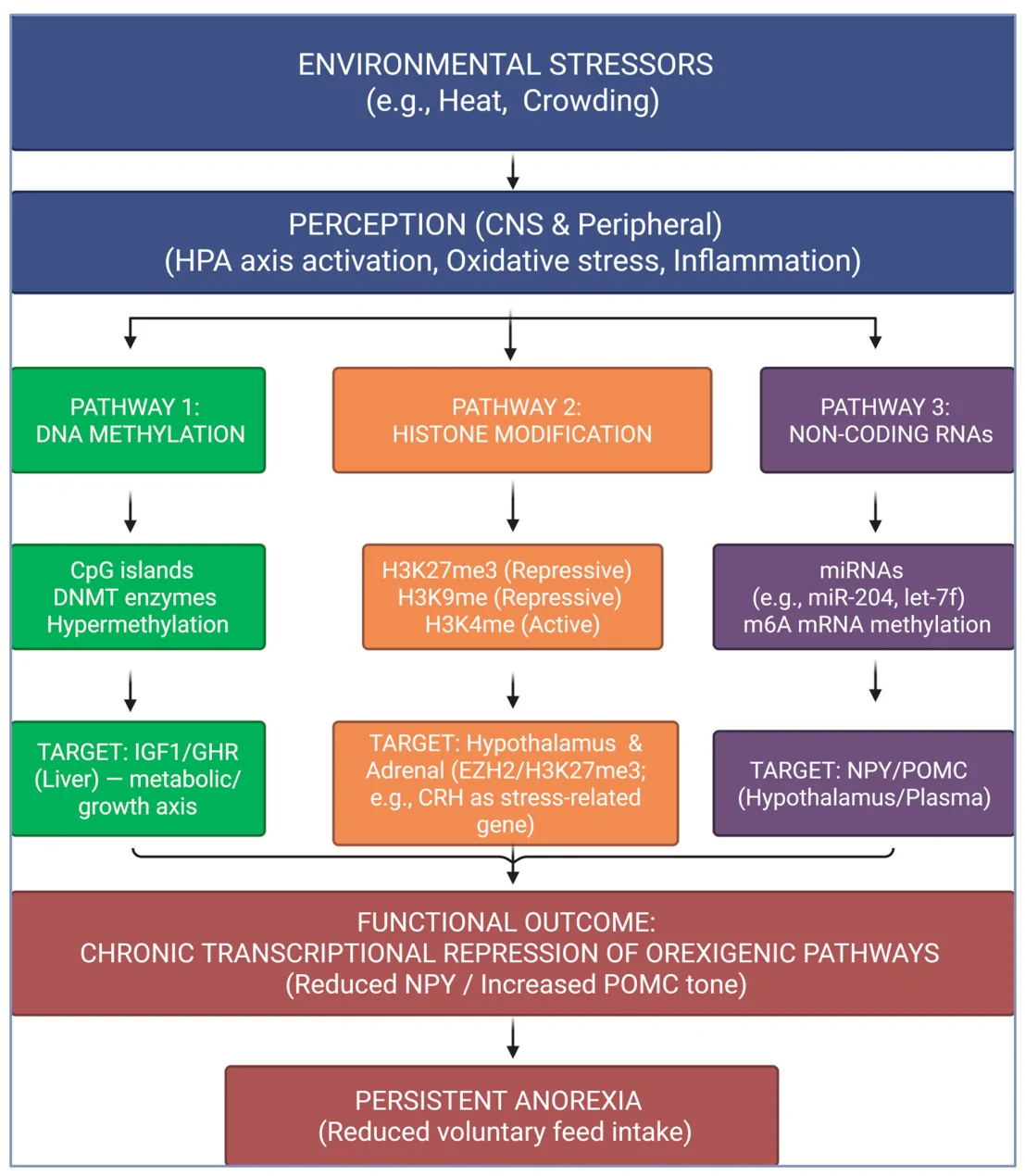
The epigenetic mechanisms that connect environmental stressors to persistent anorexia in chickens are illustrated in a schematic. The central nervous system and peripheral tissues perceive environmental stressors (e.g., heat stress, fasting, immune challenge, congestion, nutritional stress), which results in the activation of the HPA axis, oxidative stress, and systemic inflammation. Created in https://BioRender.com. Accessed on 11 October 2026. Note: CRH is an example of a stress-related gene regulated by H3K27me3, and not the direct mediator of the persistent anorexia shown downstream. Peripheral targets such as hepatic IGF1/GHR represent metabolic and growth-axis effects of stress-induced epigenetic regulation, which may influence feed intake indirectly rather than through direct modulation of hypothalamic appetite circuits.

Besides DNA modification and histone modification, new findings have also shown the importance of RNA modification, in this case N6-methyladenosine (m6A), in response to stress. This epitranscriptomic modification affects mRNA stability, splicing, translation, and nuclear export, introducing an additional layer of post-transcriptional regulation to stress-induced feeding suppression [145]. A pioneering work on broilers exposed to chronic immunological stress (by LPS injection for 10 days) showed that immune exposure markedly elevated hypothalamus m6A methylation levels. Employing MeRIP-seq (methylated RNA immunoprecipitation sequencing), researchers found 3918 differentially methylated genes, with notable enrichment in neurodevelopmental pathways such as Wnt, mTOR, and MAPK signaling. Significantly, hypothalamic expression of the orexigenic neuropeptide NPY was reduced in LPS-treated avians, alongside documented alterations in MCH expression. These transcriptional changes were associated with reduced blood serotonin (5-hydroxytryptamine; 5-HT) levels and compromised intestinal barrier function, indicating a coordinated epitranscriptomic–brain–gut axis mechanism that may contribute to immunological stress-induced anorexia [152]. This study presents the initial evidence that m6A methylation in the hypothalamus directly reacts to immunological stresses and may modulate appetite-related gene expression at the post-transcriptional level. Figure 4 shows a schematic illustrating the epigenetic processes linking environmental stresses to chronic anorexia in chickens.

## 7. Mitigation Strategies and Management

The global chicken industry is confronted with one of the most economically significant challenges: environmental stressors directly suppress feed intake and disrupt metabolic homeostasis. Nutritional interventions, environmental management, genetic strategies, and early-life programming must be integrated to mitigate these effects.

### 7.1. Nutritional Strategies

Reformulation of diets is necessary to account for decreased intake volumes as a result of decreased feed consumption during heat stress. The maintenance of caloric intake is facilitated by the increase in dietary energy density through fat supplementation, even when birds consume fewer calories. This is due to the fact that fat metabolism generates less metabolic heat than protein or carbohydrate metabolism. Numerous studies have demonstrated that nutritional strategies, including dual feeding schedules, wet feeding, and feed restriction during peak heat hours, can mitigate the detrimental effects of HS on both broiler chickens and laying hens [7]. It has been demonstrated that dietary manipulations, such as wet feeding and nocturnal feeding (which shifts feed availability to cooler periods), can mitigate the effects of HS in adult birds and chicks [11]. These methods capitalize on the natural diurnal temperature cycle, which promotes feed consumption during the lowest thermoregulatory burden.

Supplementing vitamins (particularly vitamins C and E), minerals (such as zinc, selenium, and chromium), and osmolytes (such as betaine) has been demonstrated to mitigate heat stress-induced oxidative damage and facilitate the recovery of feed intake [7,9]. An emerging class of anti-stress agents is represented by plant-derived bioactive compounds. For instance, the addition of Eucommia ulmoides extract (EUE) at a concentration of 0.8% to the diet of heat-stressed chicks resulted in a substantial increase in body weight, average daily gain, average feed ingestion, and feed conversion ratio [153]. The total antioxidant capacity (T-AOC), superoxide dismutase (SOD), and glutathione peroxidase (GSH-Px) levels were higher in the EUE groups, and the concentrations of malondialdehyde (MDA) in the serum were lower, suggesting that oxidative damage was reduced. Additionally, the efficacy of EUE in mitigating heat stress-induced tissue injury in the duodenum, heart, and stomach suggests that it could maintain gut integrity, which would be beneficial for the continuation of feed intake [153,154]. This approach has been extensively investigated and integrated into programs designed to mitigate heat stress.

### 7.2. Environmental and Housing Management

Preservation of the thermoneutral zone is achieved with open shed designs, evaporative cooling platforms, tunnel ventilation, a fogging/misting system, and proper insulation. The thermoneutral zone range is quite small for chickens, and commercial broiler chickens are especially vulnerable to heat stress, which is characterized by a higher internal heat production level in these birds [11]. The goal of any effective environmental control system is to lower the humidity and keep the ambient temperature within tolerable limits, thereby reducing the animal’s reliance on evaporative heat loss and helping it remain within its thermoneutral zone. High densities of birds create thermal stress because of the limited airflow between birds and the elevated temperature at the home’s center. Thermal stress combined with high stocking density exacerbates the negative effects on feed intake and growth performance. Mitigation necessitates the balance of economic production objectives with welfare and physiological needs [6]. The capacity of birds to endure heat during adulthood is improved by thermal conditioning during embryonic development or early post-hatch life [11]. In a study that assessed the synergistic effects of early-age feed restriction and thermal conditioning (38° C at day 5 for 24 h), it was discovered that thermal conditioning combined with feed restriction (TCFR) enhanced the physiological responses of broiler chickens in hot tropical environments [155]. This illustrates that early-life interventions can be combined to improve thermotolerance.

### 7.3. Genetic Strategies

**Figure 5:**
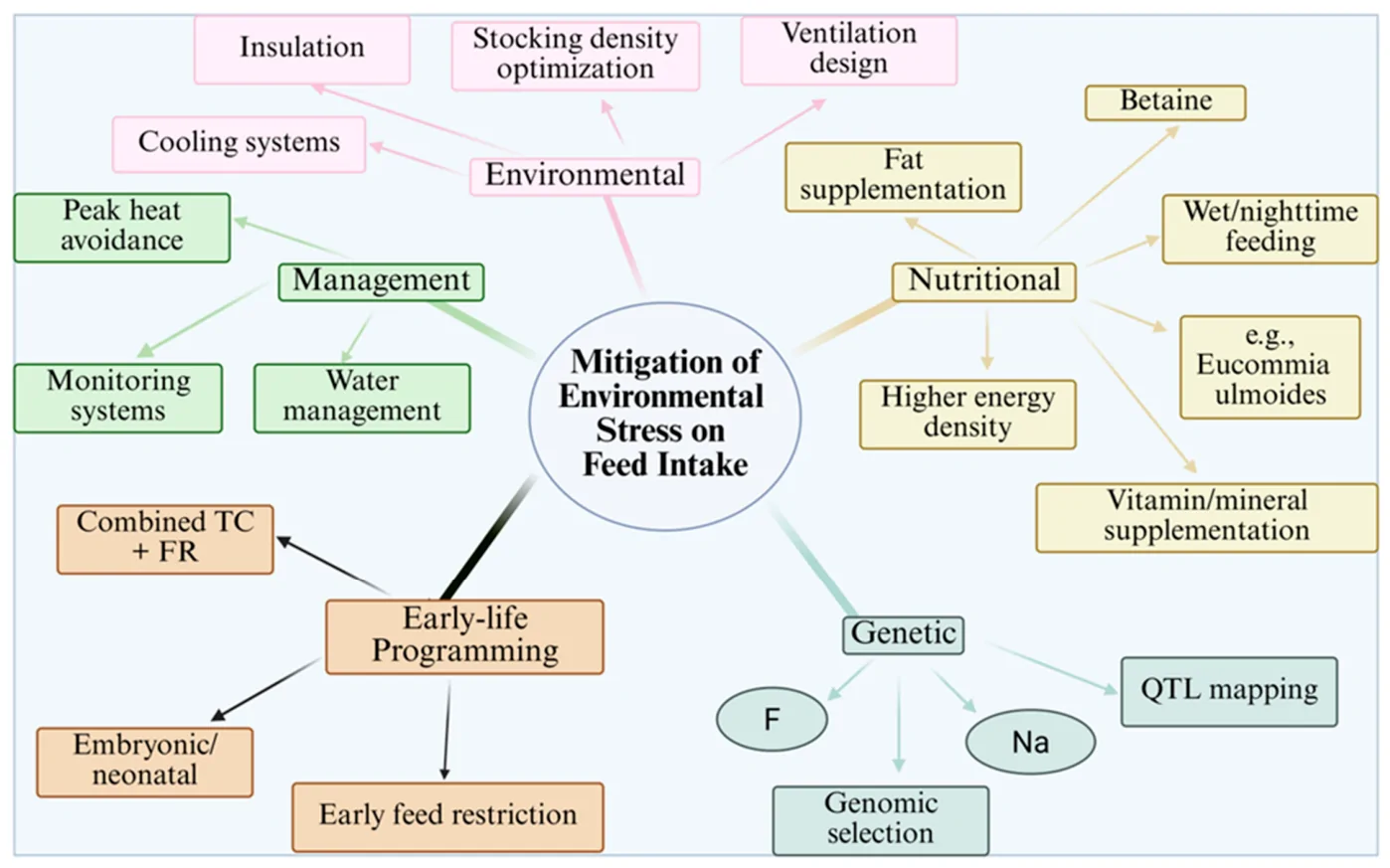
A review of techniques aimed at mitigating the reduction in feed intake caused by environmental stress. Strategies include: insulation, cooling, minimizing peak heat effects, water management, early programming (during embryonic and neonatal stages or via early feed restriction), genetic mapping and QTL identification, nutritional interventions, wet or night feeding, and monitoring systems. Created in https://BioRender.com. Accessed on 17 May 2026.

The use of the naked neck (Na) gene and frizzle (F) gene in specific breed lines has garnered significant interest as a sustainable solution. These genes diminish feather covering, so enhancing convective and radiative heat dissipation from the body surface, which subsequently lowers heat load and sustains feed intake under elevated ambient temperatures [7]. Gene mapping before breeding and genetic selection during breeding are effective methods for creating heat-resistant breeds [11]. These methods facilitate the identification of quantitative trait loci (QTLs) associated with heat tolerance, feed efficiency under stress conditions, and the accompanying physiological resilience traits. With increasing access to genomic technologies, it has become possible to integrate marker-assisted selection for heat tolerance into commercial breeding programs. Given that no single option is sufficient on its own, the most effective programs are those that combine appropriate management and nutritional strategies with genetic selection for heat tolerance. As climate change exacerbates and modifies environmental temperatures, precipitation patterns, and air conditions, the development of heat-tolerant breed lines, coupled with integrated environmental and nutritional management, is essential for sustainable chicken production. Figure 5 and Table 3 summarize the methods for mitigating feed intake reductions caused by environmental stress.

## 8. Future Direction

In spite of the progress made, there are several areas of critical need and opportunity for future research. In fact, it is now recognized that short-chain fatty acids (SCFAs) and other metabolites produced in response to stress exert epigenetic effects by changing the acetylation of histones, methylation of DNA and remodeling of chromatin in the hypothalamus and intestinal tissues [170]. The full picture of epigenetic changes in chickens that have been exposed to chronic environmental stress remains poorly understood.

Essential inquiries include:

Are stress-induced epigenetic changes in nutrition-related genes transgenerational? Evidence from in vivo thermal conditioning suggests that appetite regulation can be affected by thermal stress during embryonic development. However, whether maternal stress experienced during embryonic development also affects appetite regulation in the offspring remains to be determined. What might be the time window available to potentially reverse these epigenetic modifications caused by stress? Will targeted diet changes like histone deacetylase inhibitors and methyl donors be able to return normal feeding habits? To answer these questions, it is necessary to employ single-cell epigenomic sequencing of hypothalamic neurons and multiple-generation experimental designs. This has led to the emergence of the concept of “microbiome–gut–brain” (MGB) axis, which highlights the importance of gut microbiota composition in feed intake, consequently opening the door to a precision approach for engineering the microbiome [170,171]. Hence, future studies should be directed towards the following: the development of fiber blends and prebiotic formulations that selectively promote populations of bacteria that produce SCFAs to aid in appetite regulation under environmental stress; the identification and use of stress-resistant microbial strains that will maintain the production of beneficial SCFAs in the absence of microbial imbalance; and the exogenous administration of specific SCFAs or derivatives, bypassing the need for in situ microbial fermentation.

There is a significant difference in heat tolerance and stress resistance among different chicken breeds and genetic strains [172]. Thus, future research priorities include: the identification of QTLs and candidate genes related to sustained feed intake under HS and the use of genome-wide association studies (GWAS) and whole genome resequencing techniques; as well as the study of local and indigenous breeds adapted to tropical climates as genetic resources for appetite regulation to increase their heat tolerance potential, and the application of gene-editing technologies for example clustered regularly interspaced short palindromic repeats/CRISPR-associated protein 9 (CRISPR/Cas9) with precision to enhance some of these pathways, taking into account regulatory and ethical aspects. Finally, the improvement of understanding of the cellular responses to environmental stress should go hand-in-hand with the development of tools to assess welfare. Validation of panels of molecular stress biomarkers, including expression of the heat shock proteins, circulating CORT, oxidative stress markers and gut permeability markers, would help to provide more objective and earlier detection of feed intake suppression caused by stress before actual production loss occurs.

## 9. Conclusions

In conclusion, environmental factors significantly inhibit feed intake in chickens through complex molecular pathways that activate the HPA axis, modulate leptin and ghrelin signaling, and elevate heat shock protein levels. These interruptions in the digestive process result in low absorption, disruption of the intestinal barrier and decreased metabolic efficiency. Even though conventional management measures like insulation, cooling systems, avoiding peak heat, wet feeding and night feeding can provide partial relief, they do not directly correct the underlying biochemical imbalance; however, by reducing the initiating thermal challenge, these measures may alleviate downstream physiological disturbances. Nutritional therapies such as betaine, vitamins and minerals, and extracts of Eucommia ulmoides have shown promise in modulating the activity of stress-responsive genes and may help preserve appetite-regulating neuropeptides such as NPY and AgRP, although direct hypothalamic evidence in chickens remains limited and the underlying mechanisms are largely inferred. Epigenetic memory, as proposed, may be established during the embryonic stage and/or in response to post-hatch feed restriction, potentially contributing to long-term heat tolerance. The genes associated with resilience in feed intake under stress have been identified using a genetic approach including QTL mapping.

## Figures and Tables

**Table 1:** Stressor-induced gene expression changes affecting feed intake in chickens.

Environmental Stressor	Strain	Age	Tissue/Cell Type	Gene Expression	Feed Intake	Mechanism/Pathway	Reference
Heat Stress	Broilers, red jungle fowl/Broilers	/42 d old	Hypothalamus	↓NPY	Decrease	Reduced orexigenic signaling	[107,108]
Heat Stress	Broilers, red jungle fowl/Broilers	/42 d old	Hypothalamus	↑POMC	Decrease	Increased anorexigenic melanocortin signaling	[108]
Heat Stress	broiler (Cobb 500)	eggs	Intestinal epithelium	↓ PEPT1	Decrease	Malabsorption and reduced nutrient sensing	[109]
Feed Restriction	laying hens (Hy-line brown)	24 weeks of age	Hypothalamus	↑ NPY	Increase	Adaptive orexigenic response to energy deficit	[110]
Feed Restriction	laying hens (Hy-line brown)	24 weeks of age	Hypothalamus	↑ CART	Increase	Complex role in feeding regulation	[110]
Immune/Inflammatory Stress (LPS challenge)	yellow-feathered broilers/brown leghorn chickens	1 d old/1 d old	Liver, Intestine, Muscle	↑IL-1β, IL-3, TNF-β, IL-6	Decrease	Cytokine-induced anorexia via immune-metabolic crosstalk	[111,112]
Corticosterone	laying hens (Hy-line brown)	24 weeks of age	Hypothalamus	↓ POMC	Altered	Suppression of anorexigenic signaling	[110]
Corticosterone	laying hens (Hy-line brown)	24 weeks of age	Hypothalamus	↓ MC1R	Altered	Modulation of melanocortin system	[110]
Genetic Selection	laying and broody ducks/Red Junglefowl and White Leghorns	30-week-old/7 weeks of age	Hypothalamus	↓ CRH	Increase	Reduced anorexigenic tone promoting higher feed intake	[113]
Genetic Selection	laying and broody ducks/Red Junglefowl and White Leghorns	30-week-old/7 weeks of age	Hypothalamus	↑GR	Increase	Enhanced negative feedback on the HPA axis, reduced stress-induced anorexia	[113]
Genetic Selection	laying and broody ducks	30-week-old	Hypothalamus	↑FTO	Increase	Association with energy balance and growth traits	[110]
Heat Stress	broilers	1 d old	Hypothalamus	No change in HIF-1α	Decrease	HS affects feed intake independent of HIF-1α transcriptional changes in this context	[18]
Leptin	Broiler/Ross	3-week-old	Hypothalamus	↓NPY	Decrease	Leptin-induced anorexia via suppression of orexigenic signals	[114]
Leptin	Broiler/Ross	3-week-old	Hypothalamus	↓FAS	Decrease	Leptin-induced anorexia via metabolic signaling pathways	[114]

**Abbreviations:** CRH (Corticotropin-releasing hormone); NPY (Neuropeptide Y); POMC (Pro-opiomelanocortin); PEPT1 (peptide transporter); CART (Cocaine-and amphetamine-regulated transcript); MC1R (melanocortin 1 receptor); GR (Glucocorticoid receptor); FTO (Fat mass and obesity-associated protein); HIF-1α (Hypoxia-inducible factor 1-alpha); FAS (Fatty acid synthase); ↓ (Decrease); ↑ (Increase).

**Table 2:** Key Neuroendocrine Factors.

Hormone/Peptide	Primary Source (Chickens)	Strain	Age	Effect on Feed Intake	Key Mechanism	Reference
Ghrelin	Proventriculus	Broiler chickens Cobb/Layer White Leghorn	3 d old/8 d old	Inhibitory (unlike mammals)	CRF/HPA axis activation	[128]
Corticosterone	Adrenal gland	laying hens (Hy-line brown)	24 weeks of age	Complex (↑NPY, ↑CRH, ↑CCK, ↓POMC)	Hypothalamic gene regulation	[110]
NPY	Hypothalamus, periphery	Broiler chickens	9 d old	Stimulatory	Y1/Y5 receptor activation	[129]
POMC/α-MSH	Arcuate nucleus	Broiler chickens	1 d old	Inhibitory	MC3R/MC4R signaling	[130]
CCK	GI tract, hypothalamus	Laying hens (Hy-line brown)	24 weeks of age	Inhibitory	Vagal afferents, NTS	[110]
CRH	Hypothalamus	laying hens (Hy-line brown)	24 weeks of age	Inhibitory	Stress-appetite coupling	[110]
Spexin	Central	Broilers-Ross 308	1 d old	Inhibitory	Hypophagia induction	[131]
Adiponectin	Central	layer chickens (Hy-lines)	1 d old	Stimulatory	Orexigenic pathways	[132]

**Abbreviations:** α-melanocyte-stimulating hormone (α-MSH); Corticotropin-Releasing Hormone (CRH); Pro-opiomelanocortin (POMC); Melanocortin 3 receptor (MC3R); Melanocortin 4 receptor (MC4R); nucleus of the solitary tract (NTS); Neuropeptide Y (NPY); ↓ (Decrease); ↑ (Increase). Note: Effects of newer peptides (Spexin and adiponectin) on avian appetite are still emerging and may be context-, dose-, and route-dependent; directionality should be interpreted with caution.

**Table 3:** Summary of mitigation strategies for environmental stress-induced reductions in feed intake in chickens, with proposed neuroendocrine mechanisms, level of evidence, and representative references.

Stressor	Intervention	Poultry Type	Effect on Feed Intake	Proposed Neuroendocrine/Physiological Mechanism	Level of Evidence	Ref.
Heat stress	Dietary fat supplementation (increased energy density)	Broilers, layers	Maintains caloric intake despite reduced intake volume	Reduced diet-induced thermogenesis; indirect effect on hypothalamic energy sensing	Established (performance); mechanism inferred	[11,156]
Heat stress	Wet feeding/nocturnal feeding/dual feeding schedules	Broilers, layers	Increases voluntary intake	Aligns feeding with reduced thermoregulatory burden; reduces CRH/ACTH/corticosterone activation	Established (intake); mechanism partly inferred	[11,156,157]
Heat stress	Vitamin C, vitamin E, zinc, selenium, chromium, betaine	Broilers, layers	Restores intake	Reduces hypothalamic oxidative stress and inflammation; may preserve NPY/AgRP signaling	Moderate (intake + oxidative markers); mechanism partly inferred	[157,158]
Heat stress	Plant bioactives (e.g., *Eucommia ulmoides* extract, 0.8%)	Heat-stressed chicks	Increases average daily feed intake	Antioxidant and anti-inflammatory action; possible protection of hypothalamic appetite circuits and gut integrity	Moderate (performance + oxidative markers); mechanism inferred	[154]
Heat stress	Environmental control (tunnel ventilation, evaporative cooling, fogging, insulation)	Broilers, layers	Sustains intake	Maintains thermoneutral zone; reduces HPA axis activation	Established (intake); mechanism inferred	[7,11]
Heat stress + high stocking density	Reduced stocking density/improved airflow	Broilers	Improves intake and growth	Lowers thermal load and social stress; reduces corticosterone signaling	Established (performance); mechanism partly inferred	[7]
Heat stress	Early-life thermal conditioning (embryonic or post-hatch)	Broilers	Improves intake under later heat challenge	Programmed thermotolerance; possible epigenetic modulation of the HPA axis and appetite genes	Moderate; mechanism proposed	[11,159]
Heat stress	Early-age feed restriction + thermal conditioning (TCFR)	Broilers	Enhances physiological response and intake	Combined early-life programming of thermotolerance and metabolic regulation	Moderate; mechanism proposed	[155]
Heat stress	Naked neck (Na) and frizzle (F) genes	Broilers, layers	Sustains intake	Reduced feather cover enhances heat dissipation; lowers HPA axis activation	Established (intake); mechanism partly inferred	[156]
Heat stress	Marker-assisted selection/QTL mapping for heat tolerance	Broilers, layers	Sustains intake	Selection for thermotolerance and feed efficiency under stress; underlying neuroendocrine mechanisms largely undefined	Emerging; mechanism largely unknown	[11]
Cold stress	Increased dietary energy density; fat supplementation	Broilers	Supports intake and thermogenesis	Compensatory increase in energy intake; possible modulation of NPY/AgRP and thyroid axis	Moderate; mechanism partly inferred	[160]
Cold stress	Adequate brooding temperature and insulation	Broilers, layers	Prevents cold-induced intake reduction	Reduces HPA axis activation and sympathetic drive	Established (performance); mechanism inferred	[161]
Ventilation/air quality (NH_3_, CO_2_, dust)	Improved ventilation rates; litter management	Broilers	Restores intake	Reduces respiratory and inflammatory stress; lowers corticosterone and hypothalamic inflammation	Moderate; mechanism partly inferred	[162,163]
Stocking density (high)	Reduced density; increased feeder and drinker space	Broilers	Improves intake	Reduces social and thermal stress; lowers CORT and HPA axis activation	Established (performance); mechanism partly inferred	[164]
Stocking density (high)	Environmental enrichment	Broilers	Improves intake and welfare	Reduces chronic stress; modulates HPA axis and behavioral stress responses	Emerging; mechanism proposed	[165]
Light (long photoperiod/continuous)	Intermittent lighting programs	Broilers	Improves intake and feed efficiency	Modulates circadian and hypothalamic appetite circuits (NPY/AgRP, POMC/CART); reduces melatonin disruption	Moderate; mechanism partly inferred	[166,167]
Light (color/intensity)	Appropriate light color and intensity	Broilers, layers	Improves intake	Influences hypothalamic and retinal pathways regulating feeding behavior	Emerging; mechanism proposed	[166]
Noise stress	Sound insulation; reduced mechanical noise	Broilers, layers	May restore intake	Reduces HPA axis activation and sympathetic stress responses; mechanism largely undemonstrated in chickens	Emerging; mechanism largely unknown	[168]
Noise stress	Habituation/environmental enrichment	Broilers, layers	Improves welfare and intake	Reduces chronic stress; possible modulation of corticosterone and hypothalamic appetite signaling	Emerging; mechanism proposed	[169]

**Abbreviations:** CRH, corticotropin-releasing hormone; ACTH, adrenocorticotropic hormone; HPA, hypothalamic-pituitary-adrenal; NPY, neuropeptide Y; AgRP, agouti-related peptide; QTL, quantitative trait locus. Note on level of evidence: Grading reflects the strength and directness of available evidence linking each intervention to feed intake in chickens: Established: consistent evidence from multiple peer-reviewed studies, or at least one dedicated study or review, directly demonstrating an effect on feed intake or performance in poultry. Moderate: evidence from a limited number of studies, or evidence for effects on intake and/or associated physiological markers without direct confirmation of the proposed mechanism. Emerging: preliminary, indirect, or largely theoretical evidence, including extrapolation from other species, with the mechanism proposed or largely unknown.

## Data Availability

No new data were created or analyzed in this study. Data sharing is not applicable to this article.

## Abbreviations

11β-HSD	11β-hydroxysteroid dehydrogenase
5-HT	5-hydroxytryptamine (serotonin)
ACTH	Adrenocorticotropic hormone
ADFI	Average daily feed intake
ADG	Average daily gain
AgRP	Agouti-related peptide
AMPK	AMP-activated protein kinase
BMR	Basal metabolic rate
CART	Cocaine-and amphetamine-regulated transcript
CCK	Cholecystokinin
CORT	Corticosterone
CRF	Corticotropin-releasing factor
CRH	Corticotropin-releasing hormone
CRISPR/Cas9	Clustered regularly interspaced short palindromic repeats/CRISPR-associated protein 9
DFD	Dark, firm and dry
DMC	Differentially methylated CpG site
DNA	Deoxyribonucleic acid
EUE	Eucommia ulmoides extract
FAS	Fatty acid synthase
FTO	Fat mass and obesity-associated protein
GH	Growth hormone
GHR	Growth hormone receptor
GHS-R	Growth hormone secretagogue receptor
GR	Glucocorticoid receptor
GSH-Px	Glutathione peroxidase
GWAS	Genome-wide association study
H/L	Heterophil-to-lymphocyte ratio
HIF-1α	Hypoxia-inducible factor 1-alpha
HPA	Hypothalamic–pituitary–adrenal
HPTM	Histone post-translational modification
HS	Heat stress
HSD	High stocking density
HSP	Heat shock protein
IGF1	Insulin-like growth factor 1
IS	Indoxyl sulfate
JNK	c-Jun N-terminal kinase
LPS	Lipopolysaccharide
MAPK	Mitogen-activated protein kinase
MC1R	Melanocortin 1 receptor
MC3R	Melanocortin 3 receptor
MC4R	Melanocortin 4 receptor
MDA	Malondialdehyde
MGB	Microbiome–gut–brain
m6A	N6-methyladenosine
NF-κB	Nuclear factor kappa-light-chain-enhancer of activated B cells
NH3	Ammonia
NPY	Neuropeptide Y
NTS	Nucleus of the solitary tract
PEPT1	Peptide transporter 1
PM	Particulate matter
POMC	Pro-opiomelanocortin
PO/AH	Preoptic/anterior hypothalamus
PSE	Pale, soft and exudative
PTM	Post-translational modification
PVN	Paraventricular nucleus
QTL	Quantitative trait locus
RH	Relative humidity
RNS	Reactive nitrogen species
ROS	Reactive oxygen species
SAM	Sympatho-adreno-medullary
SCFAs	Short-chain fatty acid
SOD	Superoxide dismutase
T-AOC	Total antioxidant capacity
TCFR	Thermal conditioning combined with feed restriction
THI	Temperature–humidity index
TLR4	Toll-like receptor 4
TM	Thermal manipulation
TRH	Thyrotropin-releasing hormone
TSHB	Thyroid-stimulating hormone beta subunit
UV	Ultraviolet
VFI	Voluntary feed intake
VIP	Vasoactive intestinal peptide
